# Comparison of pulpotomy with formocresol and MTA in primary molars: a systematic review and meta- analysis

**Published:** 2008-07-10

**Authors:** Masoud Fallahinejad Ghajari, Mahkameh Mirkarimi, Mehdi Vatanpour, Mohammad Javad Kharrazi Fard

**Affiliations:** 1 *Department of Pediatric Dentistry, Dental School, Shahid Beheshti University of Medical Sciences, Tehran, Iran*; 2 *Department of Endodontics, Dental School, Islamic Azad University, and Iranian Center for Endodontic Research, Tehran, Iran*; 3 *Reserch Member, Dental Research Center, Tehran University of Medical Sciences, Tehran, Iran*

**Keywords:** Formocresol, Meta-analysis, MTA, Primary molar, Pulpotomy, Systematic review

## Abstract

**Introduction:** There are various studies looking at the effects of formocresol (FC) and mineral trioxide aggregate (MTA) on pulpotomy of primary molars. This is a systematic review of literature comparing the success rates of MTA and FC in pulpotomy of primary molars.

**Materials** **and Methods:** The study list was obtained using PubMed, EMBASE, Scopus, Science Citation Index, Iran Medex, Google Scholar, the Cochrane Library, and also some hand searches contains through dental journals approved by the Iranian Ministry of Health. Papers which met the inclusion were accepted. The quality of studies for the meta-analysis was assessed by a series of validity criteria according to Jadad's scale. Eight qualified studies met the criteria. Terms of clinical outcomes and radiographic findings were evaluated in all studies to assess clinical success and root resorption. Fixed model was applied to aggregate the data of homogenous studies. A random effect model was carried out for measuring the effect size of heterogeneous studies.

**Results:** The overall clinical and radiographic success rates based on the data suggested that MTA was superior to FC (P=0.004) with the Odds Ratio=3.535 and 95% confidence interval (1.494-8.369).

**Conclusion:** Primary molars pulpotomy with MTA have better clinical and radiographic success rates than FC. (Iranian Endodontic Journal 2008;3:45-9)

## Introduction

Maintaining the pulpally involved deciduous teeth in a healthy state until the time of normal exfoliation remains to be one of the challenges for pedodontics. Several materials have become popular as pulpotomy medicaments (1). The first time formaldehyde containing medicaments were used was in 1874, Formocresol (FC) (a mixture of equal parts of tricresol and formalin) has been used as the most common capping material for pulp fixation for many years (1,2). Success rate of FC pulpotomy has been 70-97% in the last decades. Concerns have been raised about the toxicity, potential carcinogenicity, cytotoxicity, allergenicity, systemic disturbances, and the possibility of affecting the permanent successors (2-5). FC in pulpotomy has been replaced with sulfate ferric, electro surgery, hydroxyapatite, bone morphogenic protein, laser, and mineral trioxide aggregate (MTA) (6-8). MTA is a biocompatible material which has been proposed as a potential medicaments for pulpotomy in primary teeth (1,5,7-9). In many studies, the qualities of the results are affected by lack of adequate samples, poor design and inappropriate case selection. This makes the studies unreliable and introduces limitation to the results.

**Table 1:**
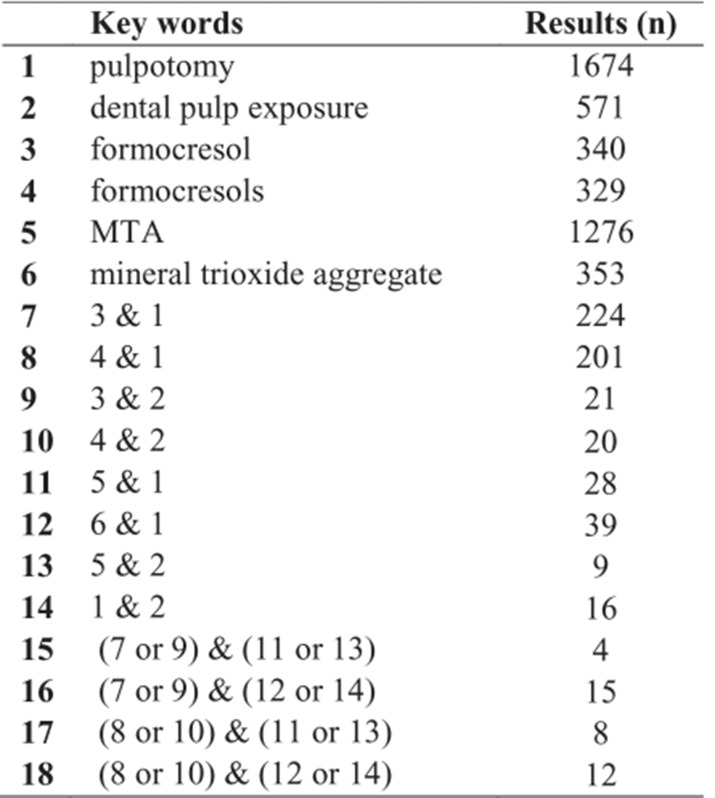
The key words used in the Medline search

In an attempt to overcome the problem of reduced statistical power, meta-analysis combines the results of several studies that address a set of related research hypotheses and can allow more accurate data analysis (10,11). The purpose of this article is applying meta-analysis to compare the clinical and radiographic success of MTA with FC as medicaments in primary molars pulpotomy.

## Materials and Methods

The study list was obtained by using PubMed (1960-March 2008), EMBASE (1984-March2008), Scopus (1996-March 2008), Science Citation Index (SCI) (1995-March 2008), Iran Medex (2004-March 2008), Google Scholar (1996-March 2008), the Cochrane library (1999- March 2008) and also hand searches through dental journals approved by the Iranian Ministry of Health. The key words used are provided in Table 1. Only those papers which met the inclusion criteria's were accepted. The search ended in March 2008 and languages of the search were limited to English and Persian. Inclusion and exclusion criteria's have been listed in Table 2. Each study was seperately assessed by at least two reviewers.

**Table 2:** Inclusion and exclusion criteria

Inclusion criteria
1- All selected teeth were human primary molars with vital pulps which have exposed by caries or trauma. 2- All selected teeth had at least 6 months follow up. 3- All selected teeth had no clinical or radiographic sign or symptoms such as internal root resorption, inter radicular or periapical bone destruction, periodontium involvement, swelling or sinus tract. 4- All selected teeth were restorable with stainless steel crowns or amalgam. 5- The outcome was evaluated by clinical symptoms and radiographic evaluation. 6- All cases were regarded as a failure when one or more of the following signs were present: internal root resorption, furcation radiolucency, periapical bone destruction, pain, swelling or sinus tract.
**Exclusion criteria**
1- Non randomized clinical trials 2- Different treatment methods 3- *In vitro*, retreat or animal studies, or histological evaluation without clinical and radiographic assessment. 4- No comparison between MTA and formocresol 5- The article could not be located.

All the studies were assessed separately and the quality of studies used for meta-analysis was evaluated by a series of validity criteria according to Jadad's scale (11). Eight randomized clinical trial studies were included. Two evaluators who were blind to authors assessed the studies independently. Over all clinical and radiographic success rates were assessed as outcome variables. The criteria for quality were based on the following:

1) Was the study randomized clinic trial?

2) Was the study described double-blind?

3) Was there a description of withdrawals and dropouts?

The scores for the first 2 questions ranged from 0 to 2 and for last question 0 to 1. The well designed studies with higher weight will exert greater influence in the meta-analysis. Results are summarized in Table 3.

## Results

**Figure1:**
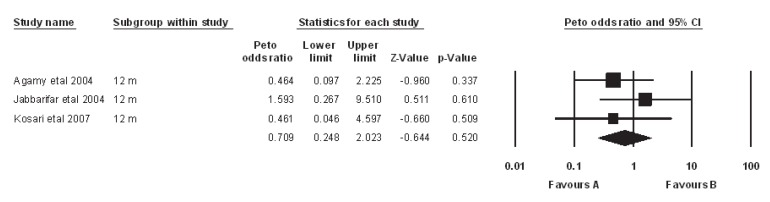
Meta-analysis (odds ratio) of studies with 12 month follow up

**Figure 2:**
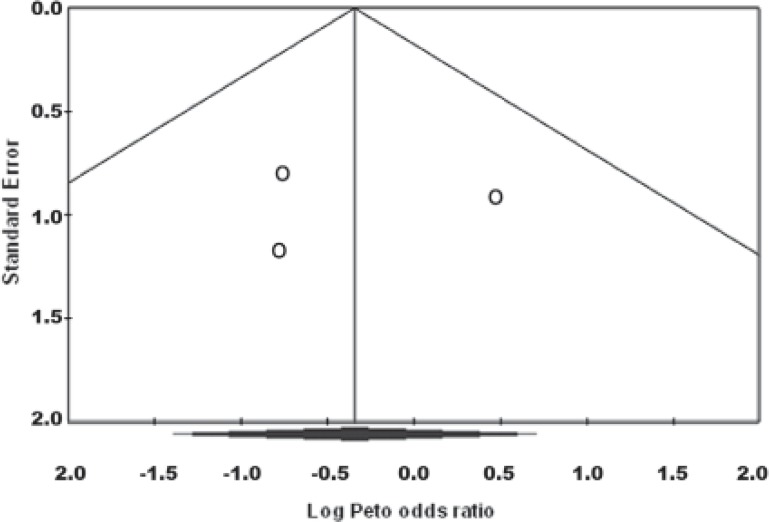
Funnel Plot of Standard Error by Peto odds ratio

***Studies with 1 year follow up:*** Heterogeneity test suggested no significant difference in overall success rate (P=0.55) than when a fixed model (Peto odds ratio) was used to aggregate data of these three studies. Findings of this meta-analysis showed that the success rate of MTA was comparable with FC and there was no significant difference between them (P=0.52) (Figure 1). Also Begg's rank correlation showed no publication bias (P=0.6) (Figure 2).

**Table 3:**
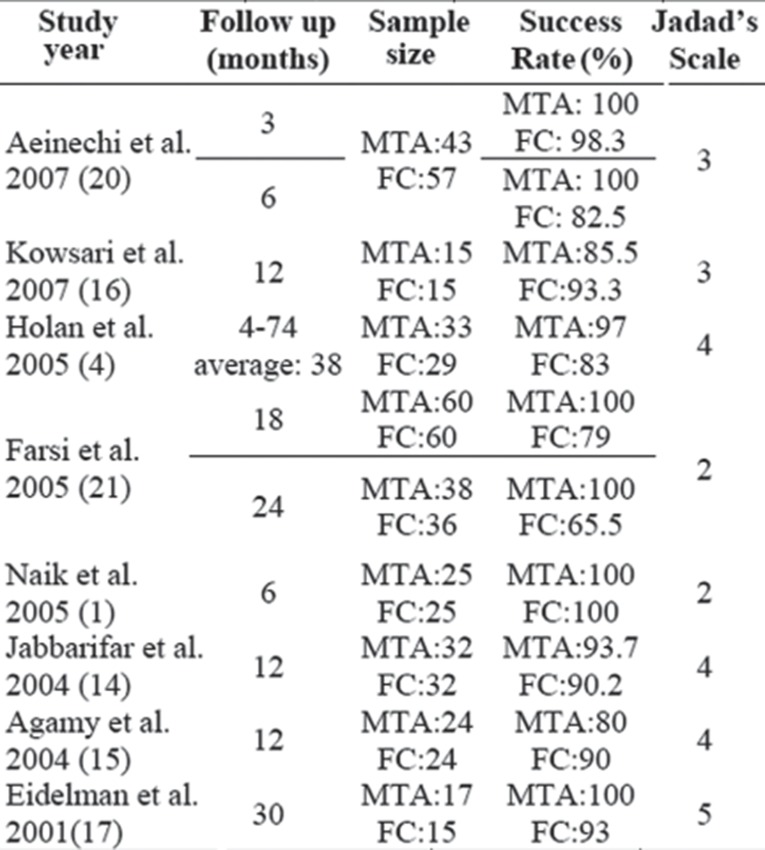
Data summary of included studies

**Figure 3:**
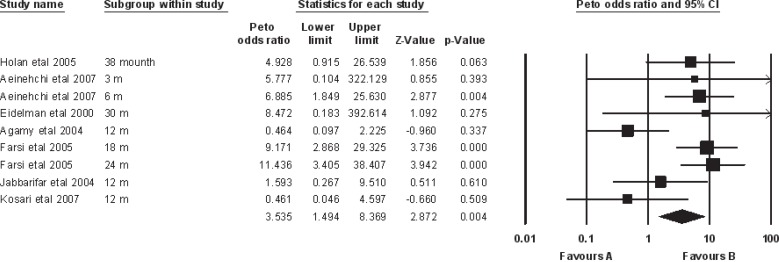
Meta-analysis (odds ratio) of all studies

**Figure 4:**
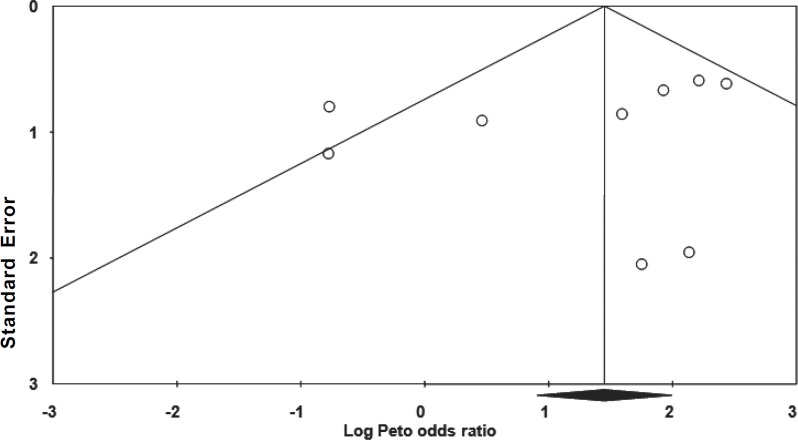
Funnel Plot of Standrad Error by Log Peto odds ratio (all studies)

***All studies: ***The results of the heterogeneity test suggests that the MTA and FC groups were significantly heterogeneous (P=0.02) next a random effect model was used to aggregate data of all included studies. Meta-analysis showed that success rate of MTA is superior to FC (P=0.004; CI: 1.494-8.369) (Figure 3). Begg's rank correlation showed no publication bias (P=0.14) (Figure 4).

**Figure 5:**
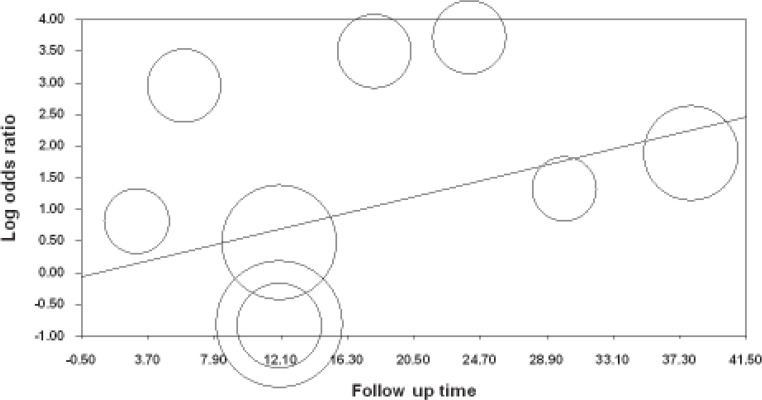
Scatter plot of meta regression of follow up times as a predictor

In comparison of two upper groups of study, it seems that time of follow up may influence the effect size of studies; therefore a meta regression analysis carried out to determine the effect of time. Results of this regression showed that the follow up intervals have no effect on the overall success rates. (P=0.96 CI: -1.56 to 1.50) (Figure 5).

## Discussion

In this review the clinical and radiographic success rates of MTA were superior to FC. MTA is a biocompatible material which provides a biological seal and has been proposed as a potential medicaments for pulpotomy in primary teeth (1,2,9,12,13).

As there are limited number of studies available and sometimes different or even controversial results, meta-analysis is a good option to provide consistent results and suggestions for clinical practice. This meta-analysis evaluated the prognosis of pulpotomy in primary molar with FC versus MTA clinically and radiographically. We have listed our findings in Table 3. All 8 studies demonstrated that the case was regarded as failure in presence of one or more of the following signs: pain, swelling, furcation radiolucency, periapical bone destruction and sinus tract. Periodontal ligament space widening was not identified as a failure in almost all studies except Jabbarifar (14) and Agamy (15). We did not include PDL widening in radiographs as a failure. Internal root resorption was identified as a failure in almost all included studies except Holan *et al*. (4).

They argued that internal root resorption should be regarded as failure only if the process reached the root's outer surface, and induced an inflammatory process in the periodontal ligament and surrounding bone. In this meta-analysis, internal root resorption was seen as a failure result. MTA used in Jabbarifar's study (14) was ProRoot MTA, Kosari *et al. *(16) used Iranian MTA, and Agamy (15) compared relative success of gray MTA and white MTA. In this meta-analysis, there was no difference between gray and white MTA.

Pulp canal obliteration (PCO) was the most common radiographic finding in both groups. PCO is the result of odontoblastic activity and suggests that the tooth is retaining some degree of vitality (17-19).

Following points affect the final degree of reliability of meta-analysis:

***A)*** The randomization method will exclude subjective interference in case selection and distribution. Except Edilman *et al.* (17), Jabbarifar *et* *al.* (14), Holan *et* *al.* (4), Aienechi *et al.* (20), and Farsi *et al.* (21) studies failed to describe their ways of randomization clearly.

***B)*** Allocation concealing, (ie, patient and outcome assessor were blinded to the treatments allocation), will guarantee an accurate assessment. This was reported only in the studies of Edileman *et al. *(17), Holan *et al. *(4), Agamy *et al. *(15), and Jabbarifar *et al. *(14).

***C)*** A small sample size will lead to decreased reliability, whereas a large one will cause the difficulties of trial control and obtaining long term data, as well as increased cost and time.

***D)*** Deciduous teeth have a shorter life span, so studies with longer follow up will be at the risk of losing case information. Longer observation periods may lead to observed lower success rates than shorter times. In the 8 studies there were different observation periods, a minimum of 6 months was required as part of the inclusion criteria in this meta-analysis.

In Pengle *et al. *(22) meta-analysis study, 6 studies were included for meta-analysis and their results concurred with ours. The pool of data was taken from different sources in Pengle's study (including the Iranian Ministry of Health approved dental journals). Moreover this information was collected two years after completing Pengle's study.

## Conclusion

Based on the present evidence, MTA can be used as a suitable replacement for FC in primary teeth especially in young children with multiple teeth requiring pulpotomy treatment.
